# Seasonal diet composition of Pyrenean chamois is mainly shaped by primary production waves

**DOI:** 10.1371/journal.pone.0210819

**Published:** 2019-01-23

**Authors:** Johan Espunyes, Jordi Bartolomé, Mathieu Garel, Arturo Gálvez-Cerón, Xavier Fernández Aguilar, Andreu Colom-Cadena, Juan Antonio Calleja, Diana Gassó, Laia Jarque, Santiago Lavín, Ignasi Marco, Emmanuel Serrano

**Affiliations:** 1 Wildlife Ecology and Health Group (WE&H), and Servei d'Ecopatologia de Fauna Salvatge (SEFaS), Departament de Medicina i Cirurgia Animals, Facultat de Veterinària, Universitat Autònoma de Barcelona, Bellaterra, Barcelona, Spain; 2 Ruminant Research Group, Departament de Ciència Animal i dels Aliments, Facultat de Veterinària, Universitat Autònoma de Barcelona, Bellaterra, Barcelona, Spain; 3 Office National de la Chasse et de la Faune Sauvage, Unité Ongulés Sauvages, Gières, France; 4 Facultad de Ciencias Pecuarias, Universidad de Nariño, Pasto, Colombia; 5 Department of Ecosystem and Public Health, Faculty of Veterinary Medicine, University of Calgary, Calgary, Alberta, Canada; 6 Departamento de Biología Animal, Biología Vegetal y Ecología, Botánica, Universitat Autònoma de Barcelona, Bellaterra, Spain; 7 CREAF, Cerdanyola del Vallès, Spain; Fred Hutchinson Cancer Research Center, UNITED STATES

## Abstract

In alpine habitats, the seasonally marked climatic conditions generate seasonal and spatial differences in forage availability for herbivores. Vegetation availability and quality during the growing season are known to drive life history traits of mountain ungulates. However, little effort has been made to understand the association between plant phenology and changes in the foraging strategies of these mountain dwellers. Furthermore, this link can be affected by the seasonal presence of livestock in the same meadows. The objective of this work was to study the seasonal changes in diet composition of Pyrenean chamois (*Rupicapra p*. *pyrenaica*) and its relationship to primary production trends in a Mediterranean alpine environment. Moreover, diet composition in two populations with contrasting livestock pressure was compared in order to study the effect of sheep flocks on the feeding behaviour of chamois. From 2009 to 2012, monthly diet composition was estimated by cuticle microhistological analysis of chamois faeces collected in the eastern Pyrenees. The primary production cycle was assessed by remote sensing, using the Normalized Difference Vegetation Index. Additionally, the diet of sheep sharing seasonally the subalpine and alpine meadows with chamois was analysed. Diet selection of chamois and sheep and their overlap was also assessed. Our results show an intra-annual variation in the diet composition of Pyrenean chamois and demonstrate a strong relationship between plant consumption dynamics and phenology in alpine areas. In addition, *Calluna vulgaris*, *Cytisus* spp. and *Festuca* spp., as well as forbs in the summer, are found to be key forage species for Pyrenean chamois. Furthermore, this study couldn’t detect differences between both chamois populations despite the presence of sheep flocks in only one area. However, the detection of a shift in the diet of chamois in both areas after the arrival of high densities of multi-specific livestock suggest a general livestock effect. In conclusion, Pyrenean chamois are well adapted to the variations in the seasonal availability of plants in alpine habitats but could be disturbed by the seasonal presence of livestock. Due to the key plants in their diet, we suggest that population management programmes should focus on the preservation of mixed grasslands composed of patches of shrubs and herbs. The effects of climate change and shrub expansion should be studied as they may potentially affect chamois population dynamics through changes in habitat composition and temporal shifts in forage availability.

## Introduction

The alpine ecosystems are some of the most seasonal biomes in the world, covering around 3% of the earth’s surface area [1]. Alpine landscapes remain free of snow for a short period of time (60–120 days on average [1, 2]), limiting not only opportunities for vegetation growth but also the availability of food resources for primary consumers [3,4]. Outside this short green period, annual plants become dry and nutrient-poor [5] and the evergreens develop frost-resistance mechanisms, which in turn reduce their palatability [6]. In such extreme environments, herbivores must decide which plants should be consumed, and when and to what degree, in order to maximise their reproductive potential [7,8], health [9] and ultimately survival rates [10].

Diet selection, therefore, plays a central role in herbivore-plant interactions, with consequences for plant community composition and, in turn, the herbivore's population viability [11]. Surprisingly, little effort has been devoted to addressing this link between plant phenology and changes in the foraging strategies of mountain species whereas plant phenology and resource availability have been repeatedly shown to affect multiple fitness components in large herbivores [9,12,13]. Diet selection studies can also contribute to identifying key forage species used to maximize body condition gains during the growing season [14], or to increase the chance of winter survival [15]. Research effort in that sense provides important clues for developing population management programmes aimed at preserving areas of special interest [16].

The Pyrenean chamois (*Rupicapra pyrenaica pyrenaica*) is a medium-sized mountain ungulate widely distributed in alpine and subalpine habitats of the Pyrenees [17]. Chamois are considered capital breeders (they store energy as body reserves to meet the high energetic costs of reproduction [18]) and intermediate feeders, capable of adapting their digestive system to woody plants or grasses depending on availability [19]. Most of the chamois’ life history traits such as nursing, weaning or body mass rely on the amount of energy stored during summer [20,21]. Although chamois can undergo short seasonal altitudinal migrations of no more than 10km in response to winter hardness and variations in food quality, they are usually a sedentary and over-wintering species [22,23]. Winter diets, on the other hand, compensate for the high locomotion and thermoregulation costs in snowy landscapes [24,25]. Considerable research effort has been directed towards summer [26–28] and autumn diets [29,30], whereas information on the spring and winter feeding behaviours of chamois is practically non-existent.

In most mountainous regions, humans have traditionally moved livestock up to alpine pastures during summer to take advantage of the nutritious growing plants [31]. This transitory increase in the number of ungulates can be huge (e.g., more than 300.000 livestock units in Switzerland [32]) and may result in an overgrazing of the most palatable plant species [33]. As a result, livestock can become a potential competitor for native mountain ungulates by equally using the same finite resources (scramble competition [34]), depleting the availability of a resource for native species (exploitation competition) or directly disturbing the behaviour of these species [35]. The consequences of such interactions between ecologically close ungulate species constitute a major challenge for community and foraging ecologists [36]. In European mountains, for example, chamois shares habitat with livestock during summer, mainly horses, cattle and sheep [35]. Because of their morphological and functional similarities, resource competition between chamois and domestic sheep (*Ovis aries*) has been the most studied by far [37–39]. In fact, flocks of sheep seem to force chamois to graze in suboptimal areas [39–41], decreasing the proportion of highly digestive forbs in their diets [40] and probably affecting body mass gains during summer.

In this work, we describe the seasonal changes in diet composition of Pyrenean chamois and their relationship to primary production trends, using three years of monthly faecal sampling in two populations from the Catalan Pyrenees (north-eastern Spain). In particular, we describe the seasonal feeding behaviours of chamois through faecal cuticle microhistological analysis and assess the cycles of primary productivity through the NDVI (Normalized Difference Vegetation Index). As foraging behaviour of northern ungulates is presumed to be mainly determined by primary production cycle [42,43], we expected that chamois adapt their feeding strategy to plant phenology. We will also explore the effects of livestock on such seasonal feeding behaviour by studying the diet composition of chamois in two areas with different livestock communities (i.e., with and without flocks of sheep). Due to the impact of sheep flocks on chamois behaviour [38], we expected to observe a use of suboptimal resources by chamois during the cohabitation period in the area where sheep was present. Diet selection of chamois and sheep in July (i.e., taking into account plant availability), and their overlap is also assessed and discussed in order to understand the potential interactions between these species.

## Material and methods

### Study area

**Fig 1:**
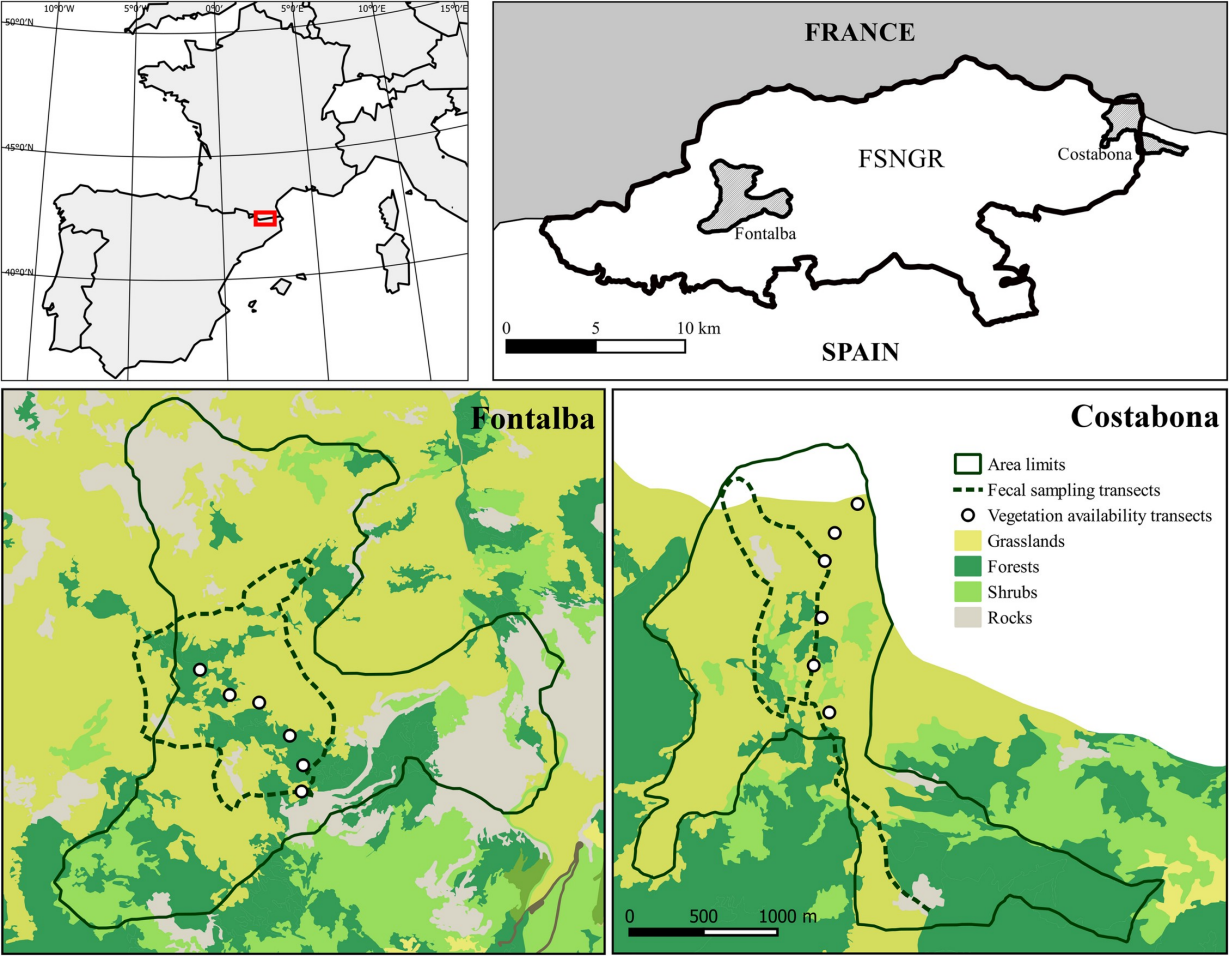
Location and map of the two study areas (Fontalba and Costabona) in the Freser-Setcases National Game Reserve. Transects for faecal sampling (dotted lines) and for vegetation availability assessment (black circles) are shown.

The study was conducted in the Freser-Setcases National Game Reserve (FSNGR), eastern Pyrenees, Catalonia, Spain (42° 22’ N, 2° 09’ E, Fig 1). The FSNGR is a mountainous area of 20.200 ha where subalpine and alpine ecosystems predominate with an average altitude of 2000 m. a. s. l. (1200–2910 m.a.s.l. at Puigmal peak).

Sampling was carried out in two areas separated by 20km of rough terrain called Costabona and Fontalba (Fig 1). The former is around 410ha and located in the north-eastern part of the FSNGR and the latter is 717ha and located in the western part of the game reserve. The two areas range from 1.700 to 2.500 m.a.s.l. and are characterised by similar features in terms of vegetation composition and structure, typical of the sub-humid subalpine and alpine bioclimatic belts of the southern slopes of the Pyrenees with a noticeable climatic Mediterranean influence [44]. During the study period, annual mean temperature was 5.7°C (min = 4.68, max = 6.21) and mean yearly accumulated rainfall was 1042.4 mm (min = 762.6, max = 1282.8). From July to September, mean temperature was 12.7°C (min = -12.6, max = 12.8) and mean accumulated rainfall was 214.5 mm (min = 169.2, max = 283.0). From November to April, mean accumulated snowfall was 1124mm (min = 877, max = 1354) and mean snow depth was 128mm (min = 41.2, max = 336.7, period 2009–2012, data from Nuria meteorological station located at 1971 m.a.s.l. in the core FSNGR, Servei Meteorològic de Catalunya < www.meteocat.com >).

In Fontalba, from mid-May to late October, at least 220 chamois share habitat with cattle (n = 309) and horse (n = 47) herds. In Costabona, a group of approximately 100 chamois coexists with herds of at least 647 cattle, 71 horses and 352 sheep. Stocking rates are around 0.57 livestock units (LU)/ha in Fontalba and 2LU/ha in Costabona (S1 Table). Sporadically, other herbivores such as roe deer and mouflon were observed in the study areas but they were not taken into account in this study due to their low densities (less than 300 for the whole FSNGR in the case of mouflon) and encounter rate.

### Vegetation availability

The vegetation of the study areas mainly consists of subalpine and alpine grasslands dominated by graminoid taxa (e.g., *Festuca* and *Carex* genera) and patches of *Trifolium alpinum* and *Calluna vulgaris*. Scattered *Pinus uncinata* forest patches also grow with an understory of small woody groundcover shrubs (e.g., *Juniperus communis*, *Rhododendron ferrugineum*) [44]. The vegetation availability during June 2011 was assessed following the point-intercept method proposed by Daget & Poissonet [45]. In brief, we defined 6 transects of 10m (see Fig 1) at different altitudes in each study area (from 1900 to 2500 m.a.s.l.). In each transect, at every 10 cm interval, a 50 cm steel needle was vertically planted in the ground and all plant species touching the needle were identified and recorded (i.e. 100 points of vegetation measurement per transect). The frequency of occurrence for each plant species was then calculated by transect as: specific number of occurrences / total number of occurrences.

### Faecal sampling procedure

From the 22^nd^ of May 2009 to the 30^th^ of November 2012, fresh chamois faecal samples were collected monthly by at least two observers following defined transects of about 5 km each within the two study areas (see Fig 1), between 1900 and 2400 m.a.s.l. Observers located chamois groups using 10 x 42 binoculars and 20–60 x 65 spotting scopes. These transects encompassed the main vegetation communities and the altitudinal movement of chamois throughout the year within each study area. Once group size, composition and precise location of chamois were recorded, observers collected fresh droppings at the exact location where animals were seen and in their surroundings. Based on the colour, texture and the presence of mucus, we estimated a maximum of five hours between defecating and collection [46]. Six faecal samples per transect were collected in separated labelled plastic bags and transported to the laboratory where they were frozen at -20°C. Groups of animals that were observed twice were only sampled once in order to avoid double-sampling the same individual. For further cuticle microhistological analyses, all six samples collected along the transect were gathered into a monthly sample (86 monthly samples in total: 44 for Fontalba and 42 for Costabona). The extent of our study areas was based on the monitoring of these populations of chamois through nine years of a capture-mark-observation study (unpublished data).

In the Costabona area, sheep faecal samples were also collected monthly from June to October 2011 and 2012 following the same sampling protocol. Similarly, six faecal samples were collected along the same transect were pooled into a monthly sample (9 monthly samples in total, as in September 2012, sheep were not observed in the area and faecal samples could not be collected).

### Diet composition assessment

A cuticle microhistological analysis of chamois and sheep faecal samples was used for our diet composition assessment [47]. This non-invasive method has been widely used for studying diet composition of both wild [38,48] and domestic animals [49] without interfering with their feeding behaviour [50]. Following Stewart’s protocol [51], samples were thawed, washed with distilled water and ground in a mortar to separate the epidermal fragments. Ten grams of sample were then placed in a test tube with 5 ml of 65% concentrated HNO3. The test tubes were then boiled in a water bath for 1 min. After digestion in HNO3, the samples were diluted with 200 ml of water. This suspension was then passed through 1.00 and 0.25 mm filters. The 0.25–1.00mm fraction was spread on glass microscope slides in a 50% aqueous glycerine solution and cover-slips were fixed with DPX microhistological varnish. Two slides were prepared from each sample. Later, slides were examined by the same operator under a microscope at ×100 and ×400 magnifications and plant fragments were recorded and counted up to 200 units of leaf epidermis. An epidermis collection of 55 main plant taxa from the study area was made and used as a reference for identification of fragments in faecal samples. Since 28 plant taxa were present at levels less than 1% of the fragments, all recorded plants were pooled into five functional groups, namely: leguminous woody plants (hereafter LW), non-leguminous woody plants (NLW), graminoid plants (GR), leguminous forb plants (LF) and non-leguminous forb plants (NLF).

### Primary production assessment

We used the Normalised Difference Vegetation Index (NDVI) as a proxy for vegetation productivity and phenology in Fontalba and Costabona (for review, see [52]). We worked with MOD13Q1 NDVI data extracted from the MODIS repository (Moderate Resolution Imaging Spectroradiometer) provided by NASA [53]. NDVI time series were calculated for Costabona and Fontalba for the period from January 2009 to December 2012 with 16 day composites at a spatial resolution of 250 m. NDVI pixels (146 pixels for Fontalba and 86 for Costabona) that fell within the boundaries of the study area shape files were extracted and an average of these pixel values was calculated.

### Statistical analysis

Firstly, differences in vegetation availability between the two study areas were checked using a permutational multivariate analysis of variance (PERMANOVA). This resemblance-based permutation method allows a geometric partitioning of variation across a distribution-free multivariate dataset using distance matrices [54]. PERMANOVA is widely used in ecology to compare communities across ecological gradients [55]. In our case, the multivariate response variable was the percentage of the total count of the five plant groups (NLW, LW, Gr, NLF and LF), whereas the study area (Fontalba vs Costabona) was our fixed categorical factor. Manhattan dissimilarity index was used, as it had the highest rank-order similarity with gradient separation in our community matrix.

Seasonal changes in the diet composition of chamois, in terms of plant use, were described performing the same PERMANOVA approach using the five plant groups as response variables. Three phenological periods (called Green-up from 1 March to 30 June, Plateau greenness from 1 July to 31 August, and Senescence periods from 1 September to 28/29 February; according to Villamuelas *et al* [43]), and two contrasting livestock periods (presence of livestock from June to October and absence of livestock from November to May) and their two-way interaction with the study areas were used as explanatory variables. In this case, the Bray-Curtis dissimilarity index was used as it had the highest rank-order similarity with gradient separation in this community matrix.

The relationships between diet composition and NDVI were explored using generalised additive models (GAM), based on the cubic regression splines method. We fitted a separate GAM for each plant group (LW, NLW, Gr, NLF and LF), using NDVI values as fixed explanatory factors and the percentages of each plant group as response variable. The assumptions of normality, homoscedasticity and independence were previously checked by the residual analysis. As NDVI values correspond to 16 day composites, we selected the value with the closest date to each sampling day. This GAM analysis is commonly used to explore non-linear relationships in ecology due to its robustness and flexibility [56]. Furthermore, the phenological dates for the start of the growing season (SOS, day of the year identified as having a consistent upward trend in the NDVI time series) and the peak of production (POP, corresponding with the day of the year when the NDVI reaches its maximum value in an annual time series) were also calculated. A further description of these metrics can be found in Forkel et al. (2015) [57].

Additionally, diet preferences of both chamois and sheep were explored through a plant selectivity analysis [58]. This technique compares the resources used by the animals (e.g., faecal microhistological analyses) with the resources available in the area (e.g., plant availability). In particular, a Type II selectivity analysis was performed, as information about the utilised resources is available at the individual level but available resources were assessed at the population level. Values under one indicate avoidance, values around one indicate an opportunistic consumption and values above one indicate preference.

Finally, diet overlap between Pyrenean chamois and sheep in the Costabona area was calculated using Horn’s index of overlap [59], as it is considered the method least biased by sample size when resource use is expressed as proportions [60]:

$$R_{o}=\frac{\sum (P_{ij}+P_{ik})\log\left(P_{ij}+P_{ik}\right)-\sum P_{ij}logP_{ij}-\sum P_{ik}logP_{ik}}{2log2}$$

where P_ij_ and P_ik_ are the proportions of resource i used by species j (Pyrenean chamois) and species k (sheep). Index values range from zero (no resources used in common) to one (complete overlap).

All the statistical analyses were performed using the statistical software R version 3.4.2 [61] and the significance threshold was set at 0.05. The PERMANOVA approaches were conducted with the R-package “Vegan” (version 2.4–5, [62]). GAMs were implemented using the R-package “mgcv” (Version 1.6–1, [63]) and the phenological dates were calculated with “Greenbrown (version 2.4.3 [64]) while “AdehabitatHS” (version 0.3.13, [65]) was used in the plant selectivity analysis.

### Ethics statement

All necessary permits were obtained for the described field studies. Permission to conduct research in the Freser-Setcases National Game Reserve was obtained from the reserve director. Because only faecal material was collected for the present study, no Institutional Animal Care and Use Committee (IACUC) approval was required.

## Results

### Vegetation availability

We recorded 70 plant taxa (65 different taxa in Fontalba and 65 in Costabona) with 60 of these detected in both areas. Only 13 of these plants were present at levels greater than 1% of the fragments (representing 83.2% of the total availability, See Table 1). A mean of 0.25% of the observed fragments could not be identified (min = 0%, max = 6.5%). The PERMANOVA test confirmed that plant availability was similar between our studied areas (Pseudo F_1,10_ = 0.37, p-value = 0.792).

**Table 1:** Plant availability (i.e., proportion of a given plant taxa with respect to the total of recorded plants in %) assessed by the line-intercept method in 6 transects of 10m length conducted at altitudes ranging from 1900 to 2500 m.a.s.l. in two areas (Costabona and Fontalba) in the Freser-Setcases National Game Reserve, Catalonia, north-eastern Spain. Only plants present at levels greater than 1% are represented.

Plant	Fontalba	Costabona
**Non-leguminous woody**		
*Calluna vulgaris*	7.36	7.92
*Juniperus communis*	5.12	4.62
*Pinus uncinata*	1.26	1.73
Other NLW	0.85	0.51
**Leguminous woody**		
*Cytisus spp*.	4.88	4.32
Other LW	0.69	1.12
**Graminoids**		
*Avenula pratensis*	1.26	1.88
*Carex caryophyllea*	9.63	12.44
*Festuca* spp.	33.86	32.30
*Nardus stricta*	3.58	2.49
Other Gr.	0.61	2.54
**Non-leguminous forbs**		
*Cruciata glabra*	1.42	0.66
*Hieracium pilosella*	2.68	1.12
*Plantago monosperma*	1.99	2.13
Other NLF	10.20	11.27
**Leguminous forbs**		
*Lotus corniculatus*	1.34	1.02
*Trifolium alpinum*	11.99	7.52
Other LF	1.26	4.37

Graminoids were the most abundant functional group followed by forbs and woody species in decreasing order of importance. Graminoids represented half of the vegetation cover, with *Festuca* spp. the dominant species followed by *Carex cariophyllea*. Forbs, covering almost one third of the area, were equally represented by legumes and non-legume plants. Leguminous forbs were dominated by *Trifolium alpinum* and no non-leguminous forb species could be considered as dominant. The rest were woody plants, where the most common were some dwarf shrubs (*Calluna vulgaris* and *Juniperus communis* ssp. *alpina*) and legumes *Cytisus* spp.

### Diet composition of Pyrenean chamois

**Fig 2:**
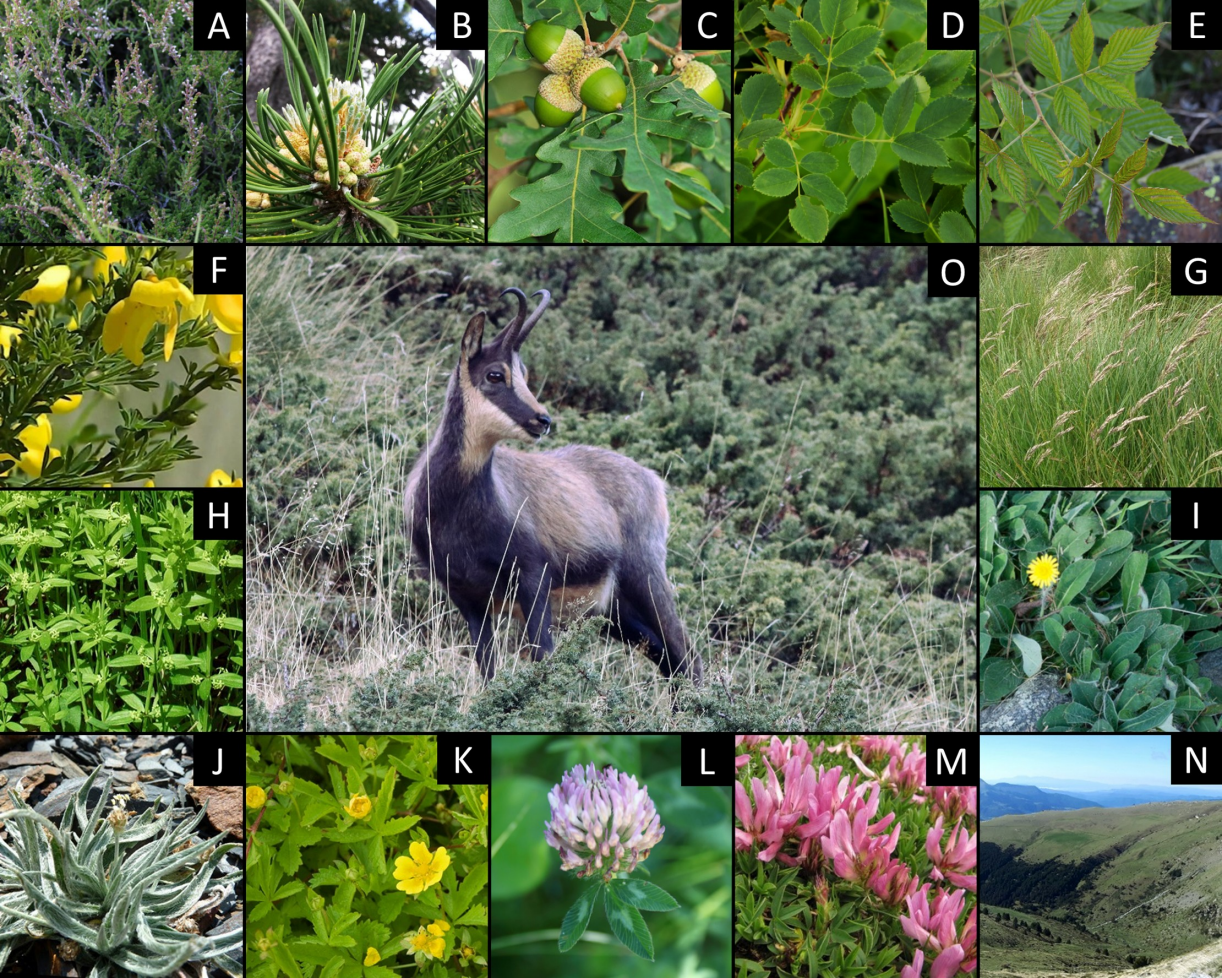
Pictures of the 13 plants most consumed by Pyrenean chamois in the Freser-Setcases National Game Reserve (FSNGR), Catalonia, north-eastern Spain. A-*Calluna vul*garis; B-*Pinus uncinata*; C-*Quercus* sp.; D-*Rosa* sp.; E-*Rubus* sp.; F-*Cytisus* spp.; G-*Festuca* spp.; H-*Cruciata glabra*; I-*Hieracium pilosella*; J-Plantago monosperma; K-Potentilla sp.; L-*Trifolium alpinum*; M-*Trifolium pratense*. Additionally, a picture of the FSNGR (N) and a Pyrenean chamois (O).

Our PERMANOVA analysis also revealed that the diet composition of Pyrenean chamois was similar between the two study areas (Pseudo F_1,85_ = 0.63, ns) and differed significantly along the three Phenological periods (Pseudo F_1,85_ = 11.83, R^2^ = 0.222, p-value = 0.001) and the two Livestock periods (Pseudo F_1,85_ = 18.26, R^2^ = 0.179, p-value = 0.001), with no significant Area x Phenological period (Pseudo F_1,85_ = 0.79, ns) or Area x Livestock period interaction (Pseudo F_1,85_ = 0.76, ns). A total of 39 plant taxa were microscopically identified in faecal samples from chamois during the three years of study and only 13 appeared in an amount greater than 1% of the total number of fragments. These 13 taxa represented 91.9% of the diet composition (Fig 2).

As we can observe in Tables 2 and 3, during the green-up period (i.e., from March to June) more than half of the diet was composed of woody species (51.6%), with *Calluna vulgaris* (21.3% of the total of fragments) and *Cytisus* spp. (17.0%) the most consumed plants. Graminoids were also an important component of the diet (32.1%) with a predominance of *Festuca* spp. (27.0%). The rest of the diet was composed of forbs (16.4%) and their consumption intensified along the months from 5.6% in March to 35.5% in June at the expanse of woody plants. *Trifolium alpinum* (3.1%) was the most consumed plant among LF, while *Plantago monosperma* (2.1%) dominated NLF.

**Table 2:** Non-leguminous woody (NLW), Leguminous woody (LW), and Graminoids (GR) plants in the annual diet composition of Pyrenean chamois from the Freser-Setcases National Game Reserve (Catalonia, north-eastern Spain), estimated by cuticle microhistological analysis of faecal samples. Values represent mean percentage of fragment frequency (min-max). Forbs content are presented in Table 3. Only plants with a consumption of more than 1% are represented.

	Green-up period				Plateau greenness		Senescence Period					
	March	April	May	June	July	August	September	October	November	December	January	February
**NLW**												
*Calluna vulgaris*	32.9 (11.5–53.0)	39.5 (20.0–53.0)	10.6 (0.0–29.5)	2.1 (0.0–6.0)	14.1 (2.5–25.0)	23.8 (8.5–44.5)	27.0 (9.5–44.0)	23.6 (10.0–33.5)	39.0 (30.5–47.0)	40.2 (35.5–45.5)	41.3 (29.0–64.5)	34.6 (22.0–46.0)
*Pinus uncinata*	11.9 (2.0–31.5)	4.5 (1.0–15.5)	2.2 (0.0–8.5)	0.9 (0.0–3.0)	0.5 (0.0–2.0)	0.4 (0.0–1.0)	0.1 (0.0–0.5)	0.2 (0.0–1.5)	1.6 (0.0–8.5)	0.4 (0.0–1.5)	0.4 (0.0–1.0)	3.0 (0.0–10.0)
*Quercus* sp.	3.3 (0.0–10.5)	2.6 (0.0–13.0)	0.0 (0.0–0.0)	0.1 (0.0–0.5)	0.0 (0.0–0.0)	0.4 (0.0–1.0)	0.9 (0.0–3.5)	3.0 (0.0–7.5)	5.1 (0.0–12.5)	9.4 (3.0–13.0)	11.1 (5.5–18.0)	7.0 (0.0–13.5)
*Rosa* sp.	0.6 (0.0–2.5)	0.2 (0.0–1.0)	1.0 (0.0–2.5)	1.9 (0.0–5.5)	1.9 (0.0–4.5)	1.4 (0.0–10.0)	2.7 (0.0–4.5)	4.1 (0.0–10.0)	0.4 (0.0–3.0)	0.0 (0.0–0.0)	0.0 (0.0–0.0)	0.2 (0.0–1.5)
*Rubus* sp.	6.1 (0.0–18.5)	7.8 (0.0–28.0)	3.1 (1.5–6.0)	5.8 (2.0–14.0)	4.1 (0.5–8.0)	6.9 (0.0–18.5)	8.3 (0.0–17.0)	13.8 (5.0–27.0)	3.3 (1.0–8.0)	3.3 (0.0–6.5)	2.6 (0.5–6.5)	3.5 (0.0–15.0)
Other NLW	0.4 (0.0–1.5)	0.4 (0.0–2.0)	0.3 (0.0–1.5)	0.3 (0.0–1.0)	0.3 (0.0–1.0)	0.0 (0.0–0.0)	0.5 (0.0–1.5)	2.3 (0.0–8.0)	0.6 (0.0–1.5)	0.2 (0.0–0.5)	0.4 (0.0–1.0)	1.4 (0.0–3.5)
Total	**55.1**	**54.9**	**17.1**	**11.1**	**20.9**	**32.9**	**39.5**	**47.0**	**50.0**	**53.5**	**55.8**	**49.7**
**LW**												
*Cytisus* spp.	24.5 (2.0–41.5)	19.6 (12.5–35.5)	9.3 (4.5–17.0)	14.8 (3.5–31.5)	24.6 (9.5–42.5)	25.6 (8.0–54.5)	25.1 (4.0–51.5)	14.8 (8.0–22.5)	15.6 (5.5–24.0)	22.0 (10.0–37.5)	24.9 (16.5–32.5)	24.1 (7.5–32.5)
Total	**24.5**	**19.6**	**9.3**	**14.8**	**24.6**	**25.6**	**25.1**	**14.8**	**15.6**	**22.0**	**24.9**	**24.1**
**GR**												
*Festuca* spp.	12.8 (4.5–21.0)	16.9 (14.5–24.5)	47.6 (34.0–60.5)	30.9 (17.5–50.0)	13.9 (6.0–18.0)	13.8 (6.0–24.0)	15.9 (7.5–27.5)	17.0 (4.5–32.5)	15.8 (8.0–25.0)	12.2 (7.0–18.0)	14.5 (11.0–22.0)	15.6 (7.5–23.5)
Other Gr.	2.0 (0.0–4.0)	6.0 (0.5–10.0)	5.9 (0.0–19.5)	6.3 (1.0–10.5)	2.1 (1.0–4.5)	1.5 (0.0–3.0)	2.6 (0.5–6.5)	1.6 (0.0–6.5)	4.1 (0.0–10.0)	3.0 (0.0–7.0)	2.0 (0.0–6.0)	2.5 (0.5–8.0)
Total	**14.8**	**22.9**	**53.5**	**37.2**	**16.0**	**15.3**	**18.5**	**18.6**	**19.9**	**15.2**	**16.5**	**18.1**

**Table 3:** Non-leguminous forbs (NLF) and leguminous forbs (LF) in the annual diet composition of Pyrenean chamois from the Freser-Setcases National Game Reserve (Catalonia, north-eastern Spain), estimated by cuticle microhistological analysis of faecal samples. Values represent mean percentage of fragment frequency (min-max). Graminoid and woody plants content are presented in Table 2. Only plants with a consumption of more than 1% are represented.

	Green-up period				Plateau greenness		Senescence Period					
	March	April	May	June	July	August	September	October	November	December	January	February
**NLF**												
*Cruciata glabra*	0.9 (0.0–5.5)	0.0 (0.0–0.0)	4.6 (1.0–15.0)	1.4 (0.0–5.5)	1.8 (0.5–4.0)	2.1 (1.0–4.0)	2.0 (0.5–4.0)	4.7 (0.0–10.5)	0.4 (0.0–2.0)	0.3 (0.0–1.0)	0.3 (0.0–2.0)	1.4 (0.0–9.5)
*Hieracium pilosella*	0.3 (0.0–1.0)	0.7 (0.0–3.0)	1.6 (0.0–3.5)	5.2 (0.0–10.5)	4.3 (0.5–17.0)	2.8 (0.0–9.0)	1.2 (0.0–4.0)	1.8 (0.0–4.0)	2.2 (0.0–11.0)	2.1 (0.0–4.5)	0.6 (0.0–1.0)	1.1 (0.0–5.5)
*Plantago monosperma*	0.1 (0.0–1.0)	0.3 (0.0–1.0)	2.4 (0.5–7.5)	5.4 (1.5–9.0)	3.1 (1.5–5.0)	2.5 (0.0–6.0)	2.1 (0.0–5.0)	0.3 (0.0–2.5)	0.9 (0.0–5.0)	0.3 (0.0–0.5)	0.1 (0.0–0.5)	0.1 (0.0–0.5)
*Potentilla* spp.	0.0 (0.0–0.0)	0.1 (0.0–0.5)	3.1 (0.5–6.5)	3.3 (1.5–6.0)	4.9 (3.0–8.5)	2.5 (0.0–6.5)	0.9 (0.0–2.5)	0.1 (0.0–1.0)	0.5 (0.0–2.5)	0.0 (0.0–0.0)	0.0 (0.0–0.0)	0.4 (0.0–3.0)
Other NLF	0.9 (0.0–3.0)	0.5 (0.0–1.0)	2.2 (0.0–6.5)	5.8 (2.5–10.0)	6.1 (2.5–14.5)	5.1 (0.0–22.5)	2.7 (0.0–7.5)	3.5 (0.0–13.0)	0.9 (0.0–4.5)	0.5 (0.0–1.5)	0.1 (0.0–0.5)	0.2 (0.0–1.0)
Total	**2.1**	**1.6**	**13.9**	**21.1**	**20.2**	**15.0**	**8.9**	**10.4**	**4.9**	**3.1**	**1.1**	**3.2**
**LF**												
*Trifolium alpinum*	2.6 (0.0–16.0)	0.0 (0.0–0.0)	2.3 (0.0–6.0)	7.5 (3.0–13.0)	7.9 (5.5–11.0)	5.9 (1.5–16.5)	5.8 (0.5–24.5)	7.4 (2.5–10.0)	7.8 (4.5–11.5)	4.8 (0.0–7.5)	1.6 (0.0–4.0)	4.2 (0.0–12.0)
*Trifolium pratense*	0.0 (0.0–0.0)	0.0 (0.0–0.0)	2.9 (1.0–7.0)	4.4 (1.5–6.0)	5.0 (2.0–8.5)	2.7 (0.5–7.0)	0.9 (0.0–2.0)	0.5 (0.0–3.5)	0.0 (0.0–0.0)	0.0 (0.0–0.0)	0.0 (0.0–0.0)	0.0 (0.0–0.0)
Other LF	0.9 (0.0–2.5)	1.0 (0.0–4.5)	1.2 (0.0–4.5)	4.5 (1.5–7.0)	5.7 (2.0–10.5)	3.0 (0.5–8.0)	1.3 (0.0–3.5)	1.4 (0.0–3.0)	1.8 (0.0–9.5)	1.5 (0.0–4.0)	0.2 (0.0–1.0)	0.6 (0.0–2.0)
**Total**	**3.5**	**1.0**	**6.4**	**16.4**	**18.5**	**11.5**	**8.1**	**9.3**	**9.6**	**6.3**	**1.8**	**4.9**

The plateau greenness period (i.e., from July to August) was still characterised by a major consumption of woody plants (52.0%), mostly *Cytisus* sp. (25.1%) and *Calluna vulgaris* (19.0%), but unlike the green-up period, forbs (32.6%) were more present than graminoids (15.6%).

Finally, during the senescence period, the consumption of woody plants reached its peak (70.3%) and the consumption of forbs reached its lowest point (11.9%). Graminoids, however, were still consumed at nearly the same rate as during the plateau greenness period (17.8%). *Calluna vulgaris* (34.3%) and *Cytisus* spp. (21.1%) were still the most consumed plants during this period, followed by *Festuca* spp. (15.2%) and *Quercus* sp. (6.1%).

### Feeding habits and primary production phenology

**Fig 3:**
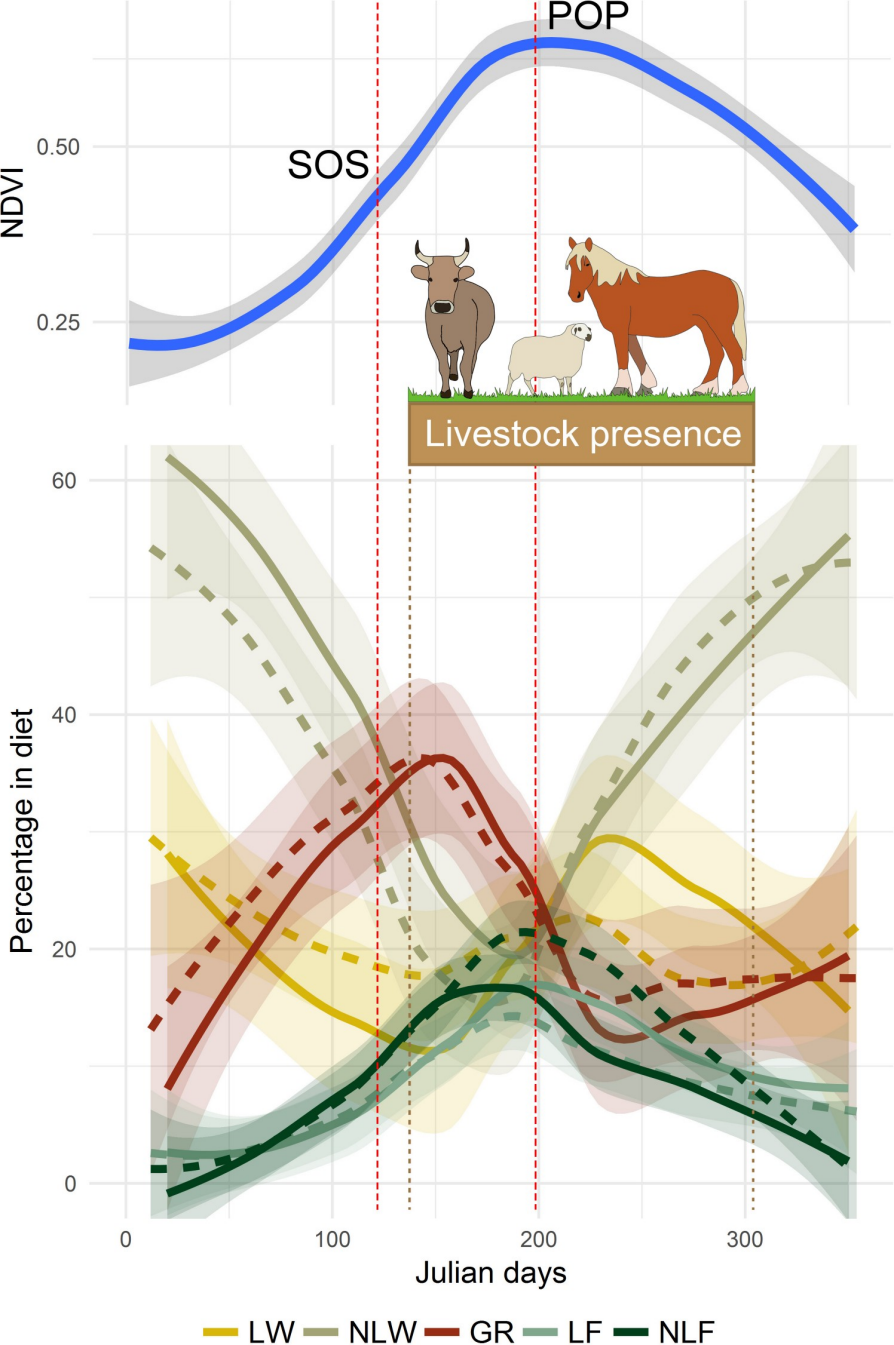
Annual relative distribution of Pyrenean chamois diet (bottom part) in Fontalba (dashed line) and Costabona (solid line) and annual NDVI trends (top part). Diet composition was assessed by cuticle microhistological analysis of faecal samples and NDVI data was extracted from the MOD13Q1 repertory. Plants are grouped into Non-leguminous woody (NLW), Leguminous woody (LW), Graminoids (GR), Non-leguminous forbs (NLF) and Leguminous forbs (LF). Start of the growing season (SOS), peak of production (POP) and presence of livestock in the area are also indicated.

In our study areas, the start of the growing season was situated on day 122 (2^nd^ of May; sd = 9.5) and the peak of production on the Julian day 199 (18th of July; sd = 10.9). As shown in Fig 3, the most consumed plants for chamois were NLW except during the green-up season, where graminoids took over NLW in synchrony with plant phenology. Graminoids took over NLW on day 120, very close to the start of the growing season and this process was reversed on day 202, very close to the peak of production (day 199).

The same synchrony appeared with forbs as the peak of consumption occurred on day 189 for NLF and 193 for LF, just a few days before the peak of production. Furthermore, NLW reached its lowest consumption on day 180. Noticeably, the increase in graminoid consumption during the growing season was stopped and reversed just after the arrival of livestock in the alpine and subalpine meadows, but one month before the primary productivity reached its peak (Fig 3).

Our GAM analysis revealed that 32.4% and 41% of the observed variability of LF (edf = 2.63; F = 11.47, p<0.005) and NLF (edf = 3.05; F = 14.46, p<0.005) were positively associated with NDVI in the study area. In contrast, NLW consumption was negatively associated with NDVI (edf = 1.85; F = 13.11, p<0.005), which explained 27.7% of the observed variability. We failed to detect any association between NDVI and either LW (variance explained: 13.3%; edf = 3.64; F = 2.22; p = 0.07) or graminoid feeding (variance explained: 1.12%; edf = 1; F = 0.95; p = 0.33).

### Resource selection of Pyrenean chamois

**Fig 4:**
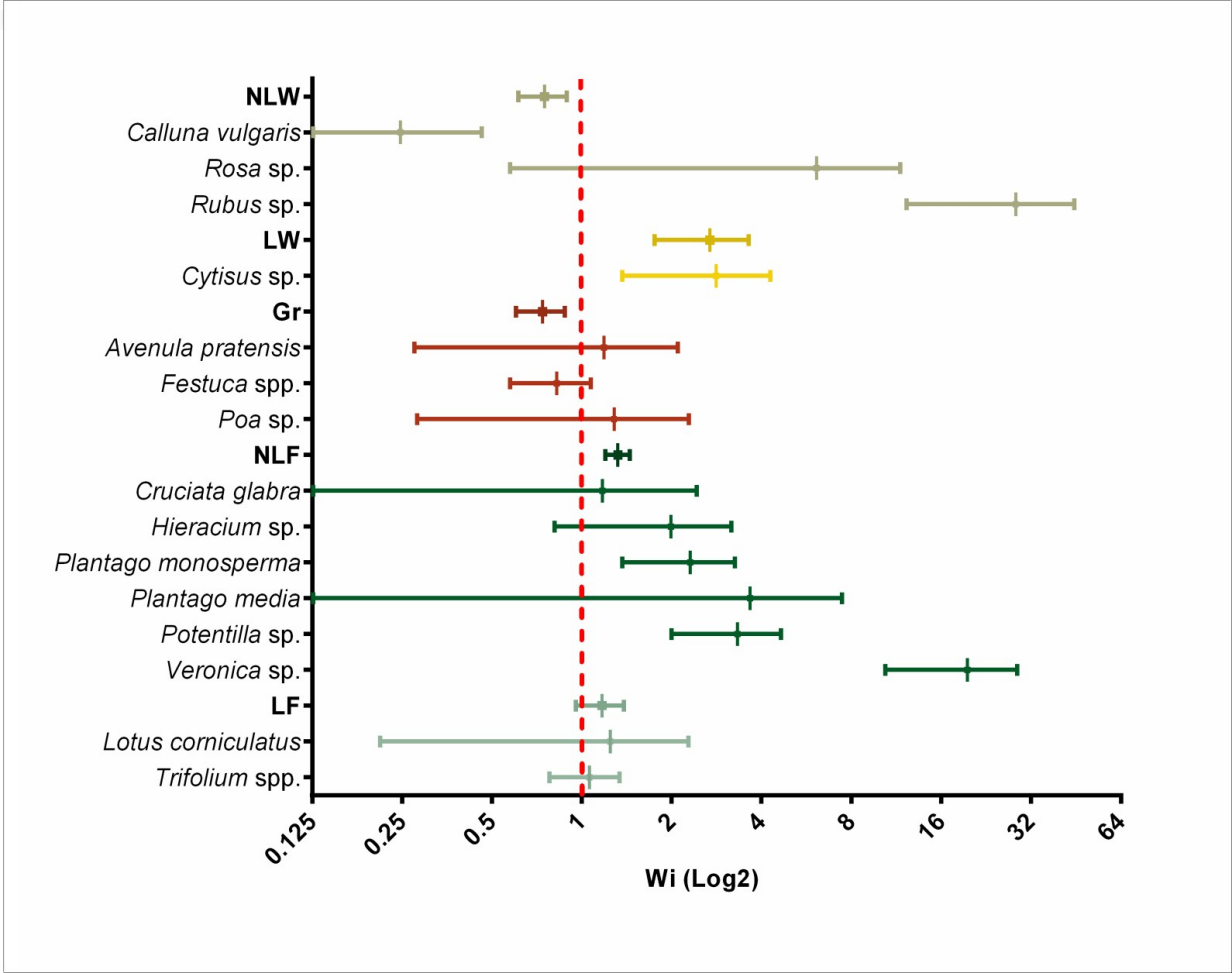
Plant selection indices (Wi) for Pyrenean chamois during June. A Type II selectivity analysis was used. Values under one (situated on the left of the red dashed line) indicate avoidance, values around one (situated around the red dashed line) indicate indifference and values above one (situated to the right of the red dashed line) indicate preference. Plant availability was assessed during June 2011 and faecal samples from chamois were collected from June 2009 to June 2012 in the Freser-Setcases National Game Reserve, Catalonia, north-eastern Spain.

As shown in Fig 4, Pyrenean chamois in June markedly preferred LW plants (Wi = 2.69, se = 0.57), showed a light preference for NLF (Wi = 1.32, se = 0.08) and LF (Wi = 1.17, se = 0.13), and a light avoidance of NLW (Wi = 0.75, se = 0.08) and GR (Wi = 0.74, se = 0.08). When plant taxa were analysed individually, Pyrenean chamois showed a strong preference for *Rubus* sp. (Wi = 28.41, se = 6.69), *Veronica* sp. (Wi = 19.55, se = 3.79), *Rosa* sp. (Wi = 6.11, se = 2.29) and *Cytisus* spp. (Wi = 2.83, se = 0.60) whereas *Calluna vulgaris* (Wi = 0.25, se = 0.09) and *Festuca* spp. (Wi = 0.82, se = 0.10) were not positively selected.

### Diet selection of sheep and diet overlap with chamois

**Fig 5:**
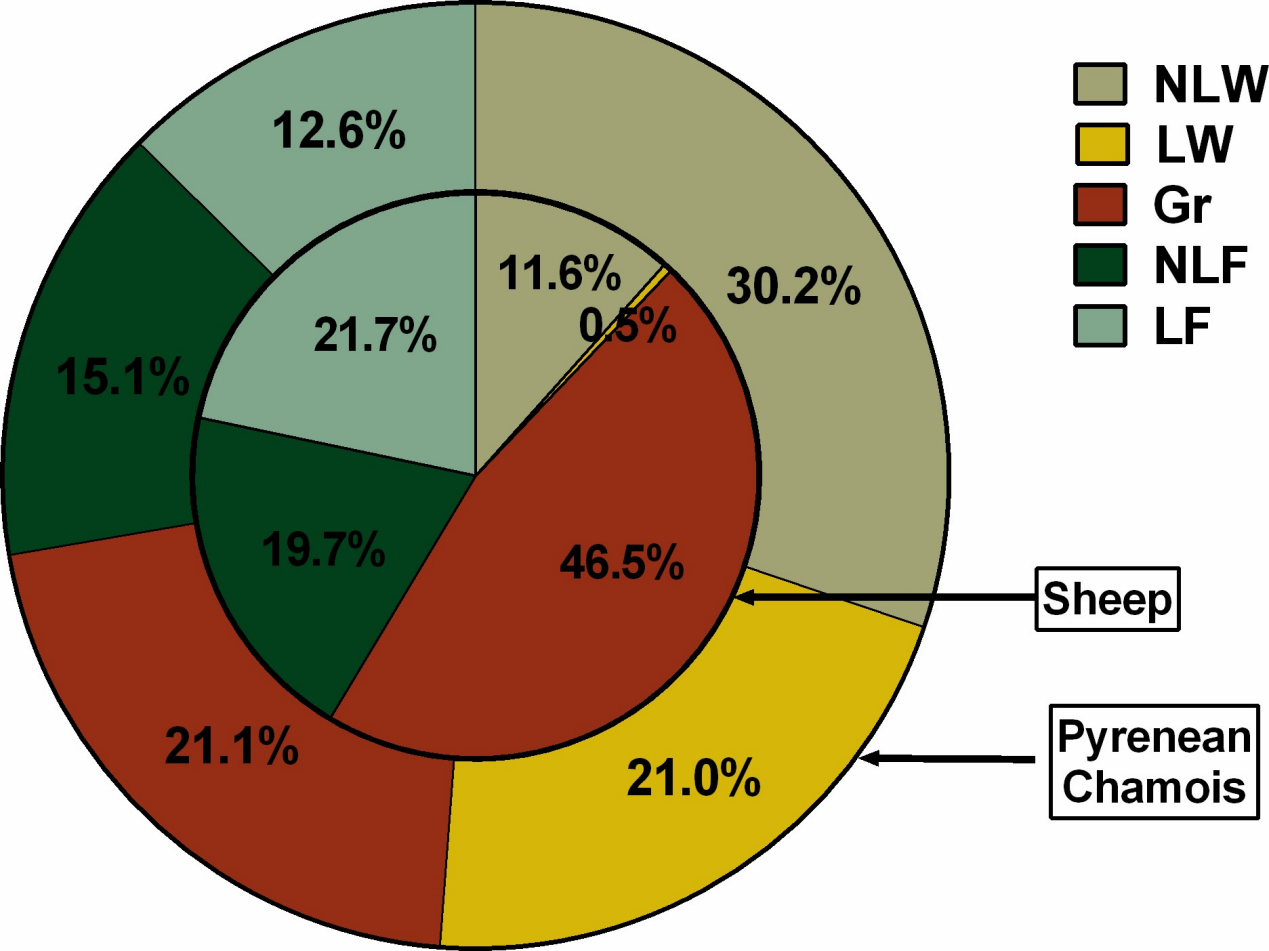
Total diet composition (in %) of sheep and Pyrenean chamois during the cohabitation period in subalpine and alpine ecosystems (from June to October). Diet composition was estimated by cuticle microhistological analysis of faecal samples collected in the Freser-Setcases National Game Reserve, Catalonia, north-eastern Spain.

The diet of sheep was assessed during the cohabitation period in subalpine and alpine ecosystems and 33 plant taxa were identified. Only 19 of these plants appeared at levels of more than 1% of fragments, which represented 95.2% of the total diet composition. A mean of 0.18% of the observed fragments could not be identified (min = 0.0%, max = 1.0%). As seen in Table 4, almost half of the diet was composed of graminoids (46.5%), with *Festuca* spp. the most important (41.1%). Forbs formed the other important part of the diet (41.4%) and were similarly divided between LF (21.7%) and NLF (19.7%), with *Trifolium alpinum* (7.4%) and *Trifolium pratense* (5.3%) the most consumed forbs. The rest of the diet was composed of woody plants (12.1%), with *Rubus* sp. (4.4%) and *Pinus uncinata* (2.7%) the most consumed. Unlike in chamois, *Calluna vulgaris* (2.1%) and *Cytisus* sp. (0.5%) were barely consumed by sheep (Fig 5). Sheep positively selected NLF and LF (Wi = 1.22, se = 0.02 and Wi = 1.5, se = 0.056) and negatively selected NLW and LW (Wi = 0.67, se = 0.17 and Wi = 0.00), while graminoids were not actively sought (wi = 0.98, se = 0.03).

**Table 4:** Diet composition of sheep in the Costabona area of the Freser-Setcases National Game Reserve (Catalonia, north-eastern Spain). **Diet composition was estimated by cuticle microhistological analysis of faecal samples. Values represent mean percentage of fragment frequency (min-max). Only plants with a consumption greater than 1% are represented.** *Faecal samples of sheep could not be collected during September 2012. Therefore, only results from September 2011 are shown.

	June	July	August	September*	October
**Non Leguminous Woody**					
*Calluna vulgaris*	0.5 (0.5–0.5)	1.5 (1.0–2.0)	3.25 (3.0–3.5)	2.0	3.0 (0.0–6.0)
*Pinus uncinata*	5.5 (4.0–7.0)	2 (2.0–2.0)	1.25 (0.5–2.0)	0.5	4.25 (3.5–5.0)
*Rosa* sp.	1.75 (1.5–2.0)	0.75 (0.5–1.0)	2.5 (2.5–2.5)	3.0	1.75 (0.0–3.5)
*Rubus* sp.	2.25 (1.0–3.5)	2.25 (1.0–3.5)	1.75 (0.0–3.5)	10.0	5.5 (3.0–8.0)
Other NLW	0 (0.0–0.0)	0 (0.0–0.0)	0 (0.0–0.0)	2.5	0.75 (0.5–1.0)
Total	**10.0**	**6.5**	**8.75**	**18.0**	**14.5**
**Leguminous Woody**					
Total	**0**	**0**	**0**	**1.5**	**0.75**
**Graminoids**					
*Festuca* spp.	44.0 (42.0–46.0)	38.0 (33.5–42.5)	28.25 (27.5–29.0)	46.5	48.5 (45.0–52.0)
*Agrostis sp*.	2.5 (2.0–3.0)	2.75 (0.5–5.0)	3.0 (2.0–4.0)	0	1.75 (1.0–2.5)
*Avenula pratensis*	3.0 (1.5–4.5)	1.5 (0.5–2.5)	1.75 (0.0–3.5)	3.5	1.25 (0.0–2.5)
Other Gr	1.0 (0.5–1.5)	2.0 (1.5–2.5)	0.5 (0.5–0.5)	2.0	1.0 (1.0–1.0)
Total	**50.5**	**44.25**	**33.5**	**52.0**	**52.5**
**Non Leguminous Forbs**					
*Hieracium pilosella*	1.0 (0.5–1.5)	3.25 (1.0–5.5)	1.5 (1.0–2.0)	3.0	1.5 (1.5–1.5)
*Plantago monosperma*	4.0 (3.0–5.0)	4.5 (3.5–5.5)	6.75 (3.5–10.0)	4.0	6.0 (6.0–6.0)
*Plantago media*	0.75 (0.0–1.5)	1.0 (1.0–1.0)	0.75 (0.5–1.0)	0	3.0 (0.5–5.5)
*Potentilla* spp.	4.5 (3.5–5.5)	6.0 (4.0–8.0)	6.25 (5.0–7.5)	2.5	3.0 (1.5–4.5)
*Ranunculus bulbosus*	5.0 (3.0–7.0)	3.75 (2.0–5.5)	3.0 (2.5–3.5)	4.0	3.0 (2.5–3.5)
*Veronica* sp.	3.0 (2.0–4.0)	1.0 (0.5–1.5)	1.5 (0.5–2.5)	0	4.25 (2.5–6.0)
Other NLF	1.0 (1.0–1.0)	1.75 (1.0–2.5)	2.25 (2.0–2.5)	1.0	0.75 (0.0–1.5)
Total	**19.25**	**21.25**	**22.0**	**14.5**	**21.5**
**Leguminous Forbs**					
*Anthyllis vulneraria*	2.75 (2.0–3.5)	4.75 (4.5–5.0)	5.75 (5.0–6.5)	6.5	1.75 (0.5–3.0)
*Chamaespartium sagittale*	0.5 (0.5–0.5)	1.75 (0.0–3.5)	2.25 (0.0–4.5)	1.0	0.75 (0.0–1.5)
*Hippocrepis comosa*	1.75 (0.0–3.5)	1.25 (0.0–2.5)	2.0 (0.0–4.0)	0	0.25 (0.0–0.5)
*Lotus corniculatus*	1.0 (1.0–1.0)	1.0 (0.0–2.0)	3.25 (0.5–6.0)	0	1.25 (0.0–2.5)
*Trifolium alpinum*	7.0 (6.5–7.5)	10.0 (9.5–10.5)	14.25 (13.5–15.0)	1.0	4.75 (4.5–5.0)
*Trifolium pratense*	6.25 (6.0–6.5)	7.0 (5.5–8.5)	6.5 (6.5–6.5)	5.0	1.5 (1.0–2.0)
Other LF	1.0 (0.5–1.5)	2.25 (1.5–3.0)	1.25 (0.5–2.0)	0.5	0.5 (0.0–1.0)
Total	**20.25**	**28.0**	**35.25**	**14.0**	**10.75**

During the cohabitating period, chamois consumed 31 out of the 33 plants consumed by sheep. The Horn’s indices of diet overlap between these two species were high (R_o_ = 0.85) at the beginning of the cohabitation period (June) and slowly decreased until September (R_o_ = 0.49), followed by a light increase in October (Ro = 0.58).

## Discussion

Our results show a clear intra-annual variation in the diet composition of Pyrenean chamois and demonstrate a strong relationship between plant consumption and phenology dynamics in our alpine area. Besides, we couldn’t detect a specific effect of sheep flocks on the diet of chamois despite a moderate to high diet overlap. However, we mark out the possibility that multi-specific livestock, and not only the presence of sheep, affect the diet of chamois during the co-habitation period.

In our study, we found that Pyrenean chamois eat a wide variety of plants and adapt their diet to seasonal changes in forage phenology, confirming that in a subalpine-alpine climate under Mediterranean influence, chamois also act as intermediate feeders. This intermediate opportunistic foraging behaviour of *Rupicapra* species has been confirmed by abounding studies [19,66–68]. In fact, depending on the floristic characteristics of their living area, they can exhibit extremely opposite dietary behaviours. Thus, chamois mainly depend on woody plants during the summer and behave as browsers in the New Zealand Alps [69], whereas they show a completely woody-free summer diet acting as grazers in the western Italian Alps [38]. This characteristic has been attributed to an anatomo-physiological adaptation of the digestive tract of this species [70].

The detection of 39 different plants in the chamois diet is within the range of other studies using the same methodology [27,29,38,71,72]. A study using DNA barcoding [30], on the other hand, showed that chamois can consume up to 110 different species. This difference has been attributed to the high level of resolution obtained with the DNA approach but also to the higher plant biodiversity of the study area (more than 1.500 species recorded, [73]). In fact, it is necessary to highlight the limitations related to dietary studies based on faecal cuticle microhistological analyses, such as ours. Even though this technique can provide reliable estimates of diet composition for herbivores [74,75], it has been noted that it tends to overestimate the less digestible species in the diet. Usually, grasses and shrubs are overdetected and easily digested forbs are underdetected [76–79]. Some forb species sporadically consumed could even be unnoticed [79]. Even if this results can vary depending on the experimental variables and the herbivore species [80,81], one must take into account the possibility of estimation bias. At the same time, when maturing, some species can become difficult to identify [77], however this was not the case in this study as the rate of unidentified species was low, even in autumn and winter. For practical purposes, microhistological analysis appears to be one of the best techniques to evaluate diet composition of large herbivores but its accuracy could be enhanced by the determination of digestibility coefficients or corrector factors at the consumer-plant level [78,82].

In our study area, a high use of woody species was observed in winter as was a high use of forbs and graminoids in spring and summer. Specifically, chamois are able to track changes in plant quality with an increasing use of NLF, LF and graminoids, in concert with the timing of spring. As a capital breeder, chamois should rely mostly on the most nutritious plants during the growing season in order to accumulate body reserves to cope with winter food scarcity and reproduction needs. By their seasonal phenology, perennials forbs and graminoids have a high protein content as early phenological growth stages of plants have a much higher nitrogen/fiber ratio than older senescent ones [5,83]. However, the dry and hot summers of our areas makes perennial plants available for only a short time. Furthermore, throughout this season, the fibre content of forbs and graminoids increases, while protein content and organic matter digestibility decrease, driving a decline in their nutritive value [84]. These results are in agreement with the findings of Galvez-Cerón et al. [23] who described a unimodal seasonal pattern of faecal nitrogen in Pyrenean chamois with a peak between May and July. This preference for herbaceous vegetation (forbs and graminoids) has also been described in several studies [19,38].

During spring and summer, chamois still consume a significant percentage of non-leguminous woody plants, mainly *Calluna vulgaris*. Except from May to August, this perennial dwarf-shrub is the plant most consumed by Pyrenean chamois in our area. The consumption of this plant is not new as it has been detected in the diet of chamois from the Cantabrian Mountains to the Italian central Alps [29,30,85]. In other areas, it even provides a permanent food source for red grouse [86] and red deer [87]. Despite having a low protein content and a high phenolic compound content compared to other plants [88], this species is rich in antioxidant compounds [89] and is annually available in the snow-free patches of grass. The quality of the winter foliage of *Calluna vulgaris* has been studied and results suggest that it has a relatively high nutritive value [90]. Furthermore, its total phenol content is lower during winter than in summer [91].

Leguminous woody plants from the *Cytisus* genus are constantly consumed across all seasons, are positively selected by chamois during summer and are the second most consumed plant taxa by chamois. *Cytisus* spp. can be found in most of the Pyrenees, Cantabrian Mountains and some parts of the Alps, where chamois is present. However, a thorough search of the relevant literature yielded that the consumption of plants in the *Cytisus* genus (and synonyms like *Genista* or *Sarothamnus*) by chamois has only been described anecdotally in a single study [30]. Although these perennial shrubs are not abundant in our areas, they form dispersed patches in the subalpine and alpine pastures and thickets in the montane abandoned meadows, and can be browsed throughout the year due to their green branches and their high height that allows them to remain uncovered by snow [92]. As with other leguminous shrubs in the Iberian mountains, even when herbaceous plants are available and despite their relatively high lignin content (up to 6% and 15% in leaves and green stems respectively [93]), *Cytisus* spp. can also represent a highly digestible and high-protein meal (up to 29% and 24% of crude protein in leaves and green stems, respectively, at the beginning of spring [93]) resulting in an interesting nutritious plant for chamois. *Cytisus spp*. are considered colonising plants [94], and thus chamois could play an important role in shrub encroachment control in open habitats caused by the abandonment of traditional farming practices [95].

*Calluna vulgaris*, *Cytissus* spp. and *Festuca* spp. are therefore key forage species for Pyrenean chamois throughout the year but forbs are equally important during the growing season. For this reason, population management programmes should focus on the preservation of mixed grasslands composed of patches of shrubs and herbs. Shrub expansion is causing a shift from herbaceous to woody plant dominance in mountainous habitats worldwide, mainly due to land use [96,97]. By reducing the availability of graminoids as well as forbs during summer, this phenomenon could affect chamois population dynamics and therefore deserves attention. Furthermore, as Pyrenean chamois adapt their diet to primary production trends, the current and future effects of climate change on the alpine environment (e.g., snow cover diminution [98], shifts in the seasonal timing of plants [99] or a shortening growing season [2]) could affect the diet of chamois and therefore their population dynamics as a result of a desynchronization between resource availability and reproduction periods or lactation needs. Pettorelli *et al*. [13] already identified that changes in plant spring phenology negatively affected the juvenile growth of other alpine ungulates.

The analysis of faeces from sheep indicated a high presence of graminoids and forbs and a moderate to high diet overlap with chamois. Sheep diet in summer has been studied in the central and western Pyrenees using faecal microhistological cuticle analysis and these other studies showed even a higher consumption of graminoids (up to 77.3%) and a lower preference for forbs and woody species than our findings [100,101]. This difference could be attributed to a different vegetation composition in these specific study areas but these data were not available. Surprisingly, there were no significant differences in the diet of chamois between the two study areas despite the presence of sheep in Costabona. In fact, modification of feeding habits and spatial segregation of chamois when flocks of sheep are present have been observed in numerous areas ([38,102] but see [103]). Even if a high diet overlap can suggest competition, it can also only indicate an overabundance of resources or consumer-specific plant part selection at the bite scale, allowing the coexistence of relatively similar large herbivores [104]. Still, in open landscapes, the real extent of resource availability at the population level can be complex to assess and some bias may occur when diet composition is compared with vegetation availability.

This study shows that, despite a high consumption of graminoids during the green-up season by chamois, there was a marked reduction in the use of this resource right after the green-up season started. This fact concurred with the arrival of livestock in the shared meadows and point out the possibility of an over-use of graminoids in the meadows where this resource is abundant. In fact, the preference for graminoids and forbs by sheep has also been described in horses and cattle [105,106] and spatial disturbance caused by large livestock flocks has been suggested by Chirichella et al. (2003; [40]). It is possible that the spatial aggregation of high densities of livestock in the meadows depleted the availability of graminoids or forced chamois to move to suboptimal patches. However, a similar study on diet composition in an area free of livestock would be necessary to exclude that the feeding behaviour of chamois is not driven by other factors such as temperature constrains or even a depletion in graminoids quality [107,108].

To conclude, chamois are well-adapted to the variations in the seasonal phenology of plants in alpine habitats. The key forage species in its diet suggest that population management plans should focus in the preservation of mixed grasslands composed of patches of shrubs and herbs. Nevertheless, climate change and shrub expansion are variables that could potentially affect chamois population dynamics through changes in habitat composition and temporal shifts in forage availability.

## Supporting information

S1 Table Maximum number of Pyrenean chamois and livestock observed in a single day during the different seasons. During monthly faecal sample transects, herbivore groups on the two study areas were located using 10 x 42 binoculars and 20–60 x 65 spotting scopes. Size and composition of groups was recorded and the total of animals from the same species observed during the day was also calculated. Maximum number of observed animals from one species in a day (in red) was used to extrapolate population numbers.
(DOCX)

S1 Dataset Data for chamois diet, sheep diet and NDVI values. (XLSX)
