# Supplementary material for: Seasonal diet composition of Pyrenean chamois is mainly shaped by primary production waves
Source: PLoS One. 2019 Jan 23;14(1):e0210819. doi: 10.1371/journal.pone.0210819 (PMC6343923; doi:10.1371/journal.pone.0210819)
Supplement: S1 Table — During monthly faecal sample transects, herbivore groups on the two study areas were located using 10 x 42 binoculars and 20–60 x 65 spotting scopes. Size and composition of groups was recorded and the total of animals from the same species observed during the day was also calculated. Maximum number of observed animals from one species in a day (in red) was used to extrapolate population numbers. (DOCX) [file pone.0210819.s001.docx]

|  |  | Fontalba | | | | Costabona | | | |
| --- | --- | --- | --- | --- | --- | --- | --- | --- | --- |
|  |  | Winter | Spring | Summer | Autumn | Winter | Spring | Summer | Autumn |
| Chamois | 2009 | - | 186 | 70 | 149 | - | 66 | 59 | 24 |
|  | 2010 | **224** | 76 | 67 | 157 | 16 | 56 | 5 | 85 |
|  | 2011 | 145 | 138 | 126 | 123 | 57 | **101** | 59 | 58 |
|  | 2012 | 133 | 108 | - | - | 15 | 19 | - | - |
| Sheep | 2009 | - | 0 | 0 | 0 | - | 0 | 257 | 54 |
|  | 2010 | 0 | 0 | 0 | 0 | 0 | 150 | 206 | 204 |
|  | 2011 | 0 | 0 | 0 | 0 | 0 | 328 | **352** | 28 |
|  | 2012 | 0 | 0 | - | - | 0 | 0 | - | - |
| Cattle | 2009 | - | 227 | 225 | 109 | - | 0 | 426 | 164 |
|  | 2010 | 0 | 15 | 179 | 146 | 0 | 235 | 224 | 185 |
|  | 2011 | 0 | 37 | **309** | 171 | 0 | 74 | **647** | 143 |
|  | 2012 | 0 | 111 | - | - | 0 | 0 | - | - |
| Horse | 2009 | - | 5 | 20 | 31 | - | 0 | 33 | 63 |
|  | 2010 | 12 | 17 | 38 | 14 | 0 | 23 | 31 | 18 |
|  | 2011 | 19 | 12 | 22 | **47** | 0 | 36 | 23 | **71** |
|  | 2012 | 11 | 11 | - | - | 0 | 0 | - | - |

**S1 Table. Maximum number of animals observed in a single day during the different seasons.** During monthly fecal sample transects, herbivore groups on the two study areas were located using 10 x 42 binoculars and 20-60 x 65 spotting scopes. Size and composition of groups was recorded and the total of animals from the same species observed during the day was also calculated. Maximum number of observed animals from one species in a day (in red) was used to extrapolate population numbers.
